# Mesoporous carbon nanomaterials in drug delivery and biomedical application

**DOI:** 10.1080/10717544.2017.1399300

**Published:** 2017-11-10

**Authors:** Qinfu Zhao, Yuanzhe Lin, Ning Han, Xian Li, Hongjian Geng, Xiudan Wang, Yu Cui, Siling Wang

**Affiliations:** a Department of Pharmaceutics, School of Pharmacy, Shenyang Pharmaceutical University, Shenyang, PR China;; b School of Chinese Materia Medica, Department of Chinese Medicinal Pharmaceutics, Beijing university of Chinese Medicine, Beijing, PR China

**Keywords:** Mesoporous carbon nanomaterials, biocompatibility, drug delivery systems, photothermal therapy, biomedical applications

## Abstract

Recent development of nano-technology provides highly efficient and versatile treatment methods to achieve better therapeutic efficacy and lower side effects of malignant cancer. The exploration of drug delivery systems (DDSs) based on nano-material shows great promise in translating nano-technology to clinical use to benefit patients. As an emerging inorganic nanomaterial, mesoporous carbon nanomaterials (MCNs) possess both the mesoporous structure and the carbonaceous composition, endowing them with superior nature compared with mesoporous silica nanomaterials and other carbon-based materials, such as carbon nanotube, graphene and fullerene. In this review, we highlighted the cutting-edge progress of carbon nanomaterials as drug delivery systems (DDSs), including immediate/sustained drug delivery systems and controlled/targeted drug delivery systems. In addition, several representative biomedical applications of mesoporous carbon such as (1) photo-chemo synergistic therapy; (2) delivery of therapeutic biomolecule and (3) in vivo bioimaging are discussed and integrated. Finally, potential challenges and outlook for future development of mesoporous carbon in biomedical fields have been discussed in detail.

## 1. Introduction

Among the carbon-family nanomaterials, sp2 carbon-based materials such as graphene, fullerene, carbon nanotube and carbon dots attract wide-spread attention for their promising performance in biomedical field including drug delivery, chemo-photothermal synergistic therapy, gene transfection and *in vivo* real-time imaging (Doane & Burda, 2012; Wei et al., 2012; Master et al., 2013; Taratula et al., 2013) due to their distinctive physiochemical, for example easily modified surface, photothermal conversion capacity, supramolecular π–π stacking and biological behaviors such as ideal compatibility, high adsorption capability and theranostic functions (Huang et al., 2011; Zhao et al., 2012c; Wang et al., 2015a). For example, both carbon nanotube (Kim et al., 2009; Moon et al., 2009; Zhou et al., 2009; Robinson et al., 2010; Wang et al., 2012b, 2014a) and graphene (Yang et al., 2010; Robinson et al., 2011; Zhang et al., 2011; Ma et al., 2012; Yang et al., 2012a; Akhavan & Ghaderi, 2013) possess ideal photothermal conversion ability within the near-infrared (NIR) region, which could be explored as photothermal candidates for synergistic treatment. Fortunately, these sp2-based carbon nanomaterials are nontoxic and biocompatible at adequate doses, proved by preclinical biodistribution, biocompatibility and hemocompatibility experiments.

Over the past few decades, mesoporous nanomaterials especially mesoporous silica nanoparticles (MSNs) have gained fast development and excellent achievements, since their large surface area, tunable pore size and high pore volume could offer large storage for guest molecules (Sheng et al., 2014; Bai et al., 2015). Compared with sp2-based carbon nanomaterials (Polizu et al., 2006; Vaijayanthimala & Chang, 2009; Ai et al., 2013; Chung et al., 2013; Kruss et al., 2013) and traditional MSNs, mesoporous carbon nanoparticles (MCNs) (Kim et al., 2008; Singh & Lillard, 2009; Chen et al., 2010; Wang et al., 2012a; Zhou et al., 2015) integrate both the advantages of MSNs and carbonaceous composition including: (1) large surface area and pore volume which are favorable for drug loading; (2) adjustable pore structure enables better control of drug release; (3) easily-modified surface could facilitate controlled and targeted drug delivery to enhance the therapeutic efficacy and reduce side effects; (4) excellent heat conversion capacity in the near- infrared (NIR) region could provide possibilities for photothermal therapy (PTT); (5) excellent biocompatibility and physi-chemical stability; (6) supramolecular π–π stacking enables the high drug-loading capacity and sustained drug release to load aromatic drugs; (7) unique optical properties and easy combination with luminescent compounds make the *in vivo* real-time monitoring and theranostic functions possible. Thus, MCNs are considered as the next generation of platforms for drug delivery and biomedical applications.

In this review, we mainly summarize the recent evolvement in drug delivery fields based on MCNs nano-carriers, including (1) immediate drug delivery systems (IDDSs), (2) sustained drug delivery systems (SDDSs), (3) controlled drug delivery systems (CDDSs) and (4) targeted drug delivery systems (TDDSs). Apart from that, we highlight the current biomedical applications of mesoporous carbon nanomaterials, including photothermal therapy, biotherapeutic agent delivery and mesoporous carbon-assisted bioimaging. Challenges and outlook faced by MCNs are also briefly discussed in the review.

## 2. Drug delivery systems based on MCNs

### 2.1. Surface modification

The carbonaceous framework of MCNs is usually formed by calcination or hydrothermal treatment at a high temperature (Fang et al., 2010; Liu et al., 2013b). And the original nature of MCNs is highly hydrophobic. In order to obtain a hydrophilic surface and make the surface modification possible, oxidization of MCNs using a concentrated strong acid (e.g. H_2_SO_4_ and HNO_3_) is the commonly adopted strategy to improve their hydrophilicity and create functional groups (Zhu et al., 2012). Or the MCNs might be oxidized by a gentle method in the presence of the ammonium persulfate (APS) in a dilute H_2_SO_4_ solution (Xue et al., 2014; Tanaka et al., 2015). This acid oxidization treatment can create abundant functional groups (mainly the carboxyl groups) on the surface of MCNs. Then, the oxidized MCNs could be further modified for different purposes such as PEGylation, polymer coating, stimuli-responsive grafting, targeting and diagnostic imaging.

### 2.2. Immediate-released drug delivery systems

There is no doubt that oral delivery is the most convenient administration route because of its simplicity, safety and low risk of infection. Nevertheless, the clinical application of hydrophobic drugs was severely limited duo to their poor solubility and low bioavailability in the gastrointestinal (GI) tract. Recently, many kinds of mesoporous materials have been widely used as carriers for the poorly soluble drugs, such as mesoporous silica (Zhang et al., 2010b, 2012), mesoporous carbon, mesoporous hydroxyapatite (Zhao et al., 2012c) and mesoporous metallic oxide (Wang et al., 2015d). Among them, mesoporous carbon nanoparticles (MCNs) possess lower density, higher porosity and stronger adsorption ability, endowing them have higher drug-loading capacity which is extremely important for those chemical drugs with high dosage requirement to meet clinical needs.

Liu et al. (2016a) reported using an Eu^3+^/Gd^3+^-EDTA-doped hollow mesoporous carbon (HMC) to improve the oral bioavailability of an insoluble model drug carvedilol (CAR) and to achieve in vivo imaging. The drug-loading efficiency (LE%) of the carboxylated HMC (SHMC) could reach up to 73.6% by the solvent evaporation method and 47.8% by the adsorption equilibrium method. The loaded CAR showed a sustained release behavior compared to the commercial capsules of CAR, and the dissolution rate decelerated as the mesoporous shells got thicker. The pharmacokinetic results showed that the AUC_0-48 h_ and *T*_max_ of CAR after oral administration increased by 2.2- and 6.5-fold, respectively, which demonstrated that the oral bioavailability was greatly improved. A relevant comparison between MCNs and MSNs has been done by Shi et al. (Lu et al., 2007; Gu et al., 2011), they found that the LE% of camptothecin (CPT) could reach up to 17% with MCNs which was higher than that of MSNs due to the aromatic rings from the framework of MCNs. During the preparation of MCNs, the carbon source-polysaccharide (PS) which derived from glucose aqueous solution upon hydrothermal treatment will undergo a carbonization process and form the condensed ring structure of MCNs with compatible environment for CPT, resulting in the high drug loading efficiency.

Zhang et al. (2013b) recently reported that carboxylated MCNs could improve the solubility and dissolution rate of hydrophobic CAR to a great extent, thus improving the bioavailability after oral administration. The drug was in amorphous state after being loaded into the nano-scale pores and both the equilibrium solubility and the dissolution rate were dramatically improved compared with crude crystalline drug powders. In addition, the relative bioavailability (RE) of CAR improved by 179.28 ± 20.5% compared with commercial product. The permeability experiment was further conducted to investigate the mechanism underlying the absorption improvement by Wang et al. (2016). They recently reported the mechanism of uniform mesoporous carbon spheres (UMCS) used as a carrier to improve the bioavailability of insoluble drug celecoxib (CEL). The interaction, adhesion and uptake of UMCS carriers by intestinal epithelial cells were studied by the transmission electron microscopy (TEM), confocal laser scanning microscopy (CLSM) and fluorescence-activated cell sorting (FACS). The result of drug transporting through human colon carcinoma (Caco-2) cell monolayer showed that UMCS can prominently reduce the rate of drug efflux and improve the permeability of CEL. As expected, the oral bioavailability of CEL loaded into the UMCS was markedly improved compared with that of commercially available capsules due to the improved dissolution rate, promote cellular uptake and decreased the rate efflux of CEL.

Since the dissolution process plays a key role in the absorption of hydrophobic agents in the GI tract, thus factors that may affect the dissolution process are elucidated in this section.

#### 2.2.1. Drug-loading methods

Drug loading was usually carried out by three methods, including melting method, physical adsorption equilibrium and solvent evaporation. Niu et al. (2013) reported that MCNs with BET surface area of 1175 m^2^/g could improve the solubility and dissolution rate of the hydrophobic fenofibrate (FFB). Different loading methods showed a great influence on the status of drug molecules within the carrier and the *in vitro* dissolution. The melting method could achieve a higher drug loading efficiency. With this method, it was hard for the drug molecules to penetrate deeply into the pores and drug molecules were unevenly distributed within the carrier, and the surface attached FFB turned to be least stable and easily re-crystallized since there was no confinement from the pore structure. For the physical adsorption method, MCNs are soaked in a drug-containing solution, drug molecules could gradually penetrate into the pore channels until an equilibrium was reached. And the drug loaded MCNs could be collected via centrifugation. The solvent evaporation method was a combination of physical adsorption and subsequent rapid solvent evaporation. It could achieve higher LE% compared with the physical adsorption method, and less surface-bonded drugs compared to the melting method. The *in vitro* release profile showed that the dissolution rate of FFB loaded MCNs was significantly enhanced since the FFB molecules was highly dispersed within the pore structure of MCNs in a noncrystalline state and could rapidly diffuse into the surrounding dissolution medium.

#### 2.2.2. Particle size

Particle size of MCNs is thought to have a great influence on the drug release rate. Zhang et al. (2013a) have synthesized highly ordered MCNs with various sizes and compared the release behavior of the model drug simvastatin (SIM). Results have shown that the release rate was greatly enhanced with the reduction of particle size, as a consequence of reduced diffusion distance.

#### 2.2.3. Pore size

The dissolution rate could also be improved by enlarging the pore size due to the decreased diffusion hindrance. Zhao et al. (2012a) has successfully fabricated fibrous ordered mesoporous carbon (FOMC) for loading an insoluble drug celecoxib (CEL) to improve the dissolution rate and enhance the oral bioavailability. The results showed that the dissolution of CEL accelerated with increasing pore size from 4.4 nm to 7 nm. Moreover, Georgios et al. (Eleftheriadis et al., 2016) compared the dissolution rate of ibuprofen from mesoporous aerogel carbons (CAs) with different pore sizes and the results showed that the ibuprofen loaded in CAs with pore size of 20 nm released faster than that in CAs with pore size of 10 nm.

In summary, MCNs have unique features compared with other types of carriers as IDDSs. The large surface area and high pore volume enable the encapsulation of drugs with a high payload. The nanoscale pore channels could keep the drugs in an amorphous or noncrystalline state, which facilitated the drug dissolution.

### 2.3. Sustained-release drug delivery systems

Compared with IDDSs that might cause the peak-and-valley drug concentration after oral administration, sustained-release drug delivery systems (SDDSs) have the ability to achieve an extended therapeutic effect by slowly releasing the encapsulated drugs over a prolonged duration, which could reduce the frequency of administration, stabilize drug concentration levels in the blood, reduce side effects and achieve better patient compliance.

Herein, the mechanism of mesoporous carbon carriers used for SDDSs have been categorized into three groups: (1) Pore structure and channel length. Sustained drug release could be achieved by regulating the morphology and mesoporous structure of the carriers that have an important effect on the release rate of the loaded drugs. (2) Interaction force between MCNs and the loaded drug. Sustained drug release could be achieved by taking advantage of the strong interaction force such as the supramolecular π–π stacking, hydrophobic interactions and electrostatic interactions between MCNs and the loaded drug. (3) Diffusion hindrance effect. When it comes to the polymer modified carbon materials, the grafted polymers will provide diffusion hindrance for the drug release, thus enabling a more sustained drug release.

#### 2.3.1. Pore structure and channel length

As discussed for IDDSs, the physical and textural natures of MCNs may greatly influence the release rate of the loaded drugs. Herein, in order to achieve a sustained release pattern, MCNs with unconnected pore structure and relatively small pore size should be designed. Besides, Qu et. al. (Qu et al., 2006) have proved that there was a strong relationship between the release rate of the loaded drug and the particle size of nanocarrier, which was proportional to the length of pore channel. To verify this point of view, Zhao et al., (2012b) compared MCNs with different mesoporous structures to load the lovastatin (LOV). The release profile showed that the release of LOV from the FOMC with two-dimensional and longer pore channel length was slower than that from the 3-dimensional UMCS with shorter channel length, revealing that drug release rate would decrease with the extension of pore channel length.

Compared with conventional MCNs, it will be easier for the hollow mesoporous carbon nanoparticles (HMCNs) to achieve sustained drug release due to their mesoporous shell and hollow cavity. To control the drug release, HMCNs with different shell thickness from 70 nm to 130 nm were prepared by Liu et al. (2016b). And higher drug-loading capacity is another appealing characteristic of HMCNs due to the unique hollow core structure and accessible pore channels on the mesoporous shell which could act as a depot of the drugs. In addition, the drug release rate from HMCNs with thicker shell was much slower due to the longer diffusion distance which indicated that it will take more time for the drug molecules to diffuse from the channels into the surrounding medium.

#### 2.3.2. Interaction force

The strong interaction force between the vehicle and the loaded drug could delay the release of drug which therefore could be employed to design the SDDSs. The carbonaceous framework of MCNs can produce supramolecular π–π stacking interactions with aromatic drug molecules to realize the sustained release (Chen et al., 2014; Zhao et al., 2017b). Zhu et al. (2012) developed MCNs with a small diameter of 90 nm as a transmembrane delivery vehicle of doxorubicin (DOX). MCNs exhibited a high loading capacity of DOX due to the hydrophobic interactions and the supramolecular π–π stacking between DOX and MCNs. Specifically, DOX loaded MCNs (DOX@MCNs) showed a sustained release under acidic condition and physiological pH due to the strong interactions between DOX and MCNs. While the release rate of DOX@MCNs was relatively quick under acidic condition in its ionized state than that in pH 7.4 PBS. Since the microenvironment of tumor tissues is slightly acidic than the normal tissues (Crayton & Tsourkas, 2011; Chen et al., 2012; Bai et al., 2015), this pH-dependent DOX-releasing profile facilitated the tumor-specific drug release from MCNs. This smart pH-dependent drug release property of DOX@MCNs made it possible to reduce the cytotoxicity to normal tissues during the circulation in the body. In addition, the DOX loaded HMCNs showed similar and sustained release properties in the published works (Chen et al., 2014; Zhao et al., 2017b).

#### 2.3.3. Diffusion hindrance effect

The polymers modified on MCNs could change the interactions between drugs and carriers and increase the diffusion distance, thus having a major impact on the drug release rate. Therefore, more attention should be paid to the modifications on MCNs due to their key role in SDDSs. In terms of SDDSs, MCNs showed more appealing characteristics such as the small pore size, strong adsorption ability and high drug loading capacity than other drug delivery carriers, thus facilitating drug dispersion and regulating drug release rate simultaneously.

**Figure 1.**
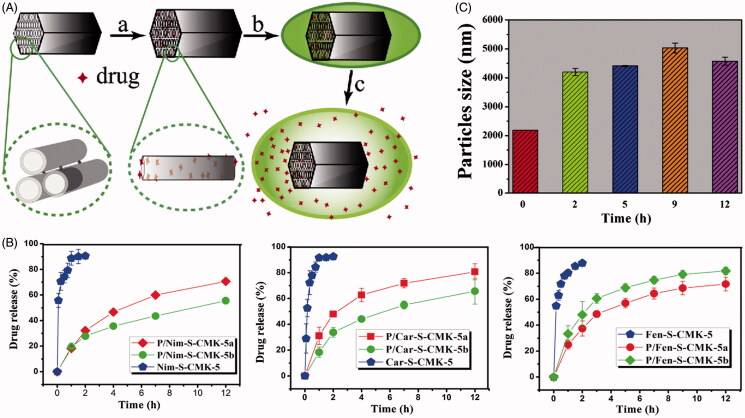
(A) Schematic diagram of the drug loading and release process of PDDA coated S-CMK-5 composites. (a) Drug solution is filled into the interior of S-CMK-5 through ultrasonication; (b) drug-loaded S-CMK-5 is added to the PDDA solution and the synthesis of composites is carried out through electrostatic interaction; (c) drug is released from the swelling PDDA coated S-CMK-5 in the release medium. (B) Nimodipine, carvedilol and fenofibrate release profiles of different drug release systems. (C) Particles size changes for P/S-CMK-5 at different sampling times in enzyme-free simulated gastric fluid (SGF, pH 1.2) (Reproduced with permission from Zhang et al., 2014b, copyright by Elsevier).

Zhang et al. (2014b) developed an oral SDDS for water-insoluble drugs, in which an organic polymer poly dimethyl diallyl ammonium (PDDA) was coated on the surface of mesoporous carbon material (CMK-5) (Figure 1(A)). Three different drugs (nimodipine, carvedilol and fenofibrate) with acidic or alkaline properties are selected as the model drugs. The release rate of three different drug from carboxylated CMK-5 (S-CMK-5) was quite fast and the cumulative drug release reached 80% within 1 h under sink conditions (Figure 1(B)). While the sustained and gradual release of drugs over a period of 12 h was observed for the PDDA coated S-CMK-5 (P/S-CMK-5). The particle size of P/S-CMK-5 was around 2 μm initially and increased to 4 μm after being kept in the release medium for 2 h and continued growing to 4.7 μm and 5 μm at 5 h and 9 h, respectively (Figure 1(C)). The increase in particle size suggested a gradual swelling of PDDA in the aqueous environment, which can effectively hinder the initial rapid drug release. These results demonstrated that the sustained release of drug was achieved mainly due to the blockage effect from the swelled polymers once contacted with the release medium. In addition, the drug showed a dramatic burst release when the concentration of salts in the release medium reached a certain level because infiltration of some salt into complex particles competed with cationic polymers and disrupted some already successfully coated PDDA polymers. Zhang et al. (Zhang et al., 2015c) developed a mesoporous carbon/lipid bilayer nanocomposites (MCLN) carrier for improved oral delivery of the poorly water-soluble drug nimodipine (NIM). The NIM-loaded MCLN exhibited a sustained drug release behavior in the simulated intestinal fluid due to the diffusion hindrance provided by the lipid bilayer. The in vivo bioavailability experiment demonstrated that the MCLN greatly enhanced the bioavailability of NIM and showed longer lasting plasma drug levels compared with the immediate-release commercial formulation.

### 2.4. Controlled/targeted drug delivery systems

#### 2.4.1. Stimuli-responsive CDDSs

In order to prevent premature drug release which would cause severe drug losses and side effects, MCNs-based CDDSs have been developed to achieve smart stimuli-responsive drug delivery systems. Such novel drug delivery systems could be achieved by modifying various ‘gatekeepers’ via covalent bonds or physical adsorption outside the pore entrance, thus preventing the drug molecule from leaking out from the drug delivery system unless being exposed to certain stimuli such as pH, redox potential, temperature and enzymes etc. The above mentioned factors could trigger the removal of the gatekeepers, then the drug begun to release in a controlled pattern. Various types of gatekeepers based on MCNs designed by researchers were summarized in Table 1.

**Table 1.** Common used gatekeepers are summarized in the below form.

Gatekeeper	Stimuli	Trigger	Modal drug	Ref.
Carbon dots	Redox	GSH	DOX	(Zhang et al., 2016b)
ZnO quantum dots	pH	Acid	Mitoxantrone	(Huang et al., 2016)
Polyaramide precursors	pH	Acid	Ibuprofen	(Sánchez-Sánchez et al., 2015)
Carbon dots	pH	Acid	DOX	(Wang et al., 2015a)
Poly(N-isopropyl acrylamide)	Temperature	Temperature	Ibuprofen	(Zhu et al., 2011)
Manganese oxide	pH/HIFU	Acid/Ultrasound	DOX	(Zhang et al., 2015b)
Polyacrylic acid	pH/Redox	Acid/GSH	DOX	(Zhang et al., 2016a)
Hyaluronic acid	Redox/Enzyme	Reducing agent /Hyaluronidase	DOX	(Zhou et al., 2015)
Hyaluronic acid	pH/ Enzyme	Acid/Hyaluronidase	DOX/Verapamil	(Wan et al., 2016)
Copper sulfide (CuS)	pH/Temperature/NIR	Acid/Temperature/NIR light	DOX	(Zhang et al., 2015a)

Among various types of stimuli-responsive drug delivery systems, pH-responsive CDDSs was the most popular due to the natural pH gradients existed between the normal and diseased tissues, and between cytoplasm and subcellular compartments (Dong & Hoffman, 1991; Jeong et al., 1997; Qiu & Park, 2001; Gupta et al., 2002; Sawant et al., 2006). It is worth mentioning that tumor tissues have more acidic microenvironment than the normal tissues (Kraus & Wolf, 1996; Trédan et al., 2007; Frérart et al., 2008; Danhier et al., 2010; Estrella et al., 2013; Justus et al., 2013). Xuan Huang et al. (2016) have developed a pH-responsive CDDS in which ZnO quantum dots were attached on the surface of MCNs as gatekeepers *via* the amide bonds and rhodamine 6 G (Rh6G) was chosen as a model drug to be loaded into the ZnO-gated MCNs (ZnO-gated MCNs@Rh6G) through the electrostatic attraction. The release profiles of Rh6G from the system showed a strong correlation with the pH values of the surrounding medium. Once the pH value was below 5.5, the opening of ‘gatekeepers’ could be induced and lead to the subsequent release of entrapped Rh6G. Interestingly, the release percentage of Rh6G increased with the decreasing of pH. Moreover, the heat generated by MCNs under near infrared radiation (NIR) would increase the local temperature which could further facilitate the release of the loaded Rh6G. The adsorptive interactions between Rh6G and pore cavities of MCNs would decrease under higher temperature and the further drop of pH could result in the decrease of electrostatic repulsion between Rh6G and the MCNs. Therefore, the combinational effects of pH and temperature could facilitate the rapid release of Rh6G from the MCNs at the tumor site once being exposed to the NIR irradiation.

Redox potential is another widely used stimulus for triggering drug release due to the significant difference in the glutathione (GSH) concentration between the extracellular fluids (2–10 μM) and intracellular (10 mM) fluids (Giri et al., 2005; Cheng et al., 2011; Liu et al., 2011a; Wen et al., 2012; Huo et al., 2014; Zhang et al., 2014a; Li et al., 2015b). Moreover, the intracellular GSH concentration in tumor cells is at least three times higher than that in normal cells which could be used an appealing intracellular stimulus (Zhao et al., 2014, 2017a). Therefore, disulfide bonds are frequently used in the design of redox-responsive CDDSs since they are stable in extracellular fluids but easily broken down in the intracellular fluid containing high concentration of GSH (Saito et al., 2003). Zhang et al. (2016b) developed a redox-responsive CDDS involving MCNs in which the fluorescent carbon dots (CDs) served as gatekeepers were grafted on the pore openings of MCNs by the cleavable disulfide bonds. In the presence of GSH, the CDs separated from MCNs due to the cleavage of disulfide bonds, thus triggering the rapid release of the encapsulated DOX. The fluorescence of CDs was quenched/‘turned off’ when grafted on the surface of MCNs, while was recovered/‘turned on’ once the CDs were detached from MCNs. Thus, the fluorescent CDs served as both a gatekeeper to realize the controlled drug release and a fluorescent probe to visualize the drug delivery process. The polymers grafted to the pore entrance could also been used as the redox-responsive gatekeepers. Li et al. (Zhang et al., 2016a) developed a poly (acrylic acid) (PAA) conjugated hollow mesoporous carbon (HMC) as a redox and pH dual-triggered drug delivery system. The PAA was conjugated on the outlets of HMC via disulfide bonds to block the drug within the mesopores of HMC. PAA was chosen for its favorable advantages, such as good biocompatibility, abundance of carboxyl groups to realize the pH-responsive drug release, extension of the blood circulation time, and the improvement of the dispersity of the nano-carriers in physiological environment.

In addition, temperature have been used as a stimulus to trigger drug release. A temperature-responsive drug delivery system composed of poly(N-isopropyl acrylamide) (PNIPAM) and the ordered mesoporous carbon (CMK-3) was developed by Shenmin Zhu et al. (2011). PNIPAM was used as a temperature-sensitive polymer due to the hydrophilic–hydrophobic transition at the ‘lower critical solution temperature’ (LCST) and has become the most widely used thermo-responsive polymer in biomedical applications. The disruption of hydrogen bonds between the PNIPAM and water could be induced when the environmental temperature reached above the LCST, which is approximately 25 °C. And the polymer chains would shrink leaving the pores of the m-CMK-PNIPAM open and ready for drug loading. However, when the temperature was below the LCST, the formation of hydrogen bonds between polymers with water molecules would lead to the swelling of polymer-chains, therefore, sealing the pores of the CMK-PNIPAM to prevent drug release.

Compared with other types of nanomaterials used in DDSs, MCNs have unique advantages. (1) The high drug-loading capacity and good dispersing stability could reduce the amount of nondrug carrier materials used to meet the therapeutic dosage needs and be administrated though different routes. (2) The tunable morphology and easily modified surface make the carbon nanomaterials perfectly suitable for constructing various types of CDDS to reduce the burst release during circulation in vivo and control the drug release upon exposed to certain stimuli. (3) The excellent heat conversion capacity in the near-infrared (NIR) region could make MCNs being used as the NIR light-responsive drug carrier for chemo-photothermal synergistic therapy, which will be discussed in detail in Section 3.1 Photothermal and synergistic therapy.

#### 2.4.2. Targeted drug delivery systems (TDDSs)

The distribution of conventional chemo-drugs is usually lack of specificity after administration which would severely limit their treatment efficacy, cause side effects and induce the multi-drug resistance (MDR) (Kartner et al., 1983; Thiebaut et al., 1987; Boesch et al., 1991; Kane, 1996; Gottesman et al., 2002; Ganta & Amiji, 2009). In the recent decades, the development of nano-DDSs has made it possible to achieve targeted drug delivery without any drug leakage during the transporting process and specifically release the drug at the tumor sites. TDDSs could reduce the high dosage demand of chemical agents and reduce the damage to normal tissues and cells to a large extent. Methods that could achieve TDDSs are mainly divided into two parts: (1) Passive targeting mechanism. Nanoparticles within the size range between 50 and 200 nm have preferential access to tumor area via the leaky vasculature which is known as enhanced permeability and retention (EPR) effect (Wisse et al., 1996). (2) Active targeting strategy, the ligands or antibodies are modified on carriers in order to endow them specificity. Particle size and hydrophilic surface play a key role in the tumor accumulation of TDDS (Yuan et al., 1995).

Wan et al. (2014) have developed a multifunctional targeted drug delivery system based on uniform mesoporous carbon spheres (UMCS), in which the branched polyethyleneimine (PEI) covalently linked with fluorescein isothiocyanate (FITC) and folic acid (FA) were grafted on the surface of UMCS. Paclitaxel (PTX) was dispersed in the mesopores of UMCS with a high LE% up to 51.37%. The results demonstrated that the FA-functionalized nanoparticles (FA–PEI–UMCS) were specifically uptaked by tumor cells with high expression of FA receptors and had superior antitumor activity compared with other PTX formulations due to a higher accumulation of FA–PEI–UMCS in tumor tissue in vivo. Moreover, the FA–PEI–UMCS nanoparticles were also used to improve the oral absorption of PTX (a BCS class IV drug) with poor oral bioavailability (Wan et al., 2015). The results demonstrated that FA–PEI–UMCS showed enhanced cellular uptake *via* FA receptor mediated endocytosis in Caco-2 cells. In addition, FA–PEI–UMCS nanoparticles significantly enhanced the permeability of PTX across the Caco-2 cell monolayer. And the in vivo results demonstrated that FA-PEI-UMCS nanoparticles not only improved the oral bioavailability of PTX, but also decreased the gastrointestinal toxicity of PTX.

#### 2.4.3. Controlled and targeted drug delivery systems

**Figure 2.**
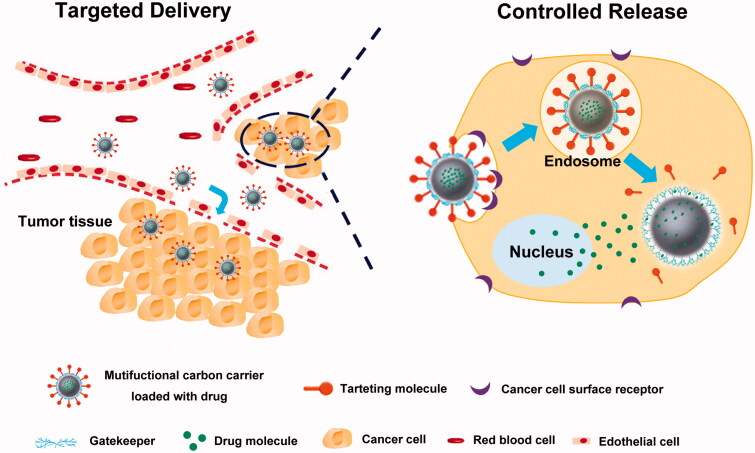
Schematic illustration for the *in vivo* process of controlled and targeted drug delivery system based on MCNs.

Recently, there is an increasing interest in the carbon-based multifunctional drug delivery systems, which can deliver drug to the target location and release the payload drug in a controlled manner to increase their cellular uptake with least premature release before reaching the target site (Figure 2). Among the various stimuli used for CDDSs, endogenous enzymes in living organisms has also emerged as a promising trigger. Wan et al. (2016) have developed a hyaluronic acid (HA) functionalized UMCS (HA-UMCS) for enzyme-responsive and targeted drug delivery. Herein, the UMCS ensured the stable encapsulation of drug in the mesopores, while the HA endowed the particles with colloidal stability, biocompatibility, cell-targeting ability and controlled drug release property. The results demonstrated that HA-UMCS were able to specifically target the CD44 receptor-overexpressing cancer cells. And the DOX and verapamil (VER) coloaded HA-UMCS (VER/DOX/HA-UMCS) exhibited a pH and hyaluronidase-1 dual-responsive release pattern which facilitated the specific drug release in the tumor microenvironment. In addition, VER/DOX/HA-UMCS exhibited a superior therapeutic effect on the HCT-116 tumor xenografted BALB/c nude mice. Zhou et al. (2015) recently designed a novel multifunctional nanoplatform based on MCNs to achieve enzyme and GSH dual-responsive drug release, targeted delivery and combinational chemo-photothermal therapy. The surface modification of MCN with biomacromolecules HA through disulfide bonds made the system be sensitive to both intracellular hyaluronidase-1 and GSH to control drug release. HA could be served as a targeting moiety towards the CD44 overexpressed cancer cells, and improve the colloidal stability and biocompatibility of MCNs, and realize the hyaluronidase-1 triggered drug release.

## 3. Biomedical applications

### 3.1. Photothermal and synergistic therapy

As a physical treatment, photothermal therapy (PTT) has been widely applied to treat cancer by using NIR-resonant nano-agents, which could absorb the NIR light and convert it into cytotoxic heat to kill the cancerous cells and reduce the invasive damage to normal cells. It is well known that the direct thermal ablation of cancer cells typically needs a high temperature (e.g. above 50 °C), which could damage the surrounding normal tissues and cells (Yang et al., 2016). Unlike the direct thermal ablation, PTT has much more mild photothermal effects, by elevating the temperature at the tumor area to 43–45 °C and maintain the hyperthermia for a few minutes, and the tumor ablation could be effectively induced. According to the literature, near-infrared (NIR) light within the wavelength range from 700 nm to 1100 nm could penetrate deeply into tissues (up to 10 cm) and is minimally absorbed by skin and tissues, which could achieve effective ablation of cancer cells (Weissleder, 2001; Wu et al., 2009; Zhang et al., 2013c).

Among various photothermal agents, MCNs are characterized by strong optical absorption in the near-infrared region, demonstrating its potential utility as the NIR-resonant nano-agent to convert the NIR light into heat and induce the ablation of cancer cells. Therefore, after being loaded with anti-cancer drugs, the MCNs was able to achieve chemo-photothermal cotherapy (Chen & Shi, 2015). Xu et al. (Xu et al., 2014) had prepared the MCNs modified with PEI and FA for the targeted drug delivery and chemo-photothermal therapy. Modification of FA could significantly enhance the uptake of drug-loaded MCNs by HeLa cells which overexpress the folate receptors. Compared with chemotherapy alone, the DOX-loaded FA/PEI/MCNs demonstrated improved treatment efficacy under the irradiation of NIR light, and the generated heat could not only lead to the apoptosis of tumor cells but also accelerate the release of DOX. Moreover, the MCNs under NIR irradiation could not only perform photothermal effects, but also show great potential in suppressing the MDR pathway.

In order to further take advantage of mesoporous carbon, HMCNs were prepared. Our group have compared the photothermal effect between MCNs and HMCNs (Li et al., 2017). Compared with the MCNs, the advantage of the HMCNs was a higher drug-loading efficiency and higher photothermal conversion efficacy due to a large hollow void and low density. Moreover, our group have also compared the heat generation efficiency and the thermal stability between HMCNs and a NIR dye IR-820 after repeated irradiations (Zhao et al., 2017b). The results demonstrated that HMCNs had excellent heat generating capacity and remained stable after exposure to NIR irradiation for three times, while the IR-820 was thermal unstable and degraded completely after exposed to NIR irradiation for only one time.

**Figure 3.**
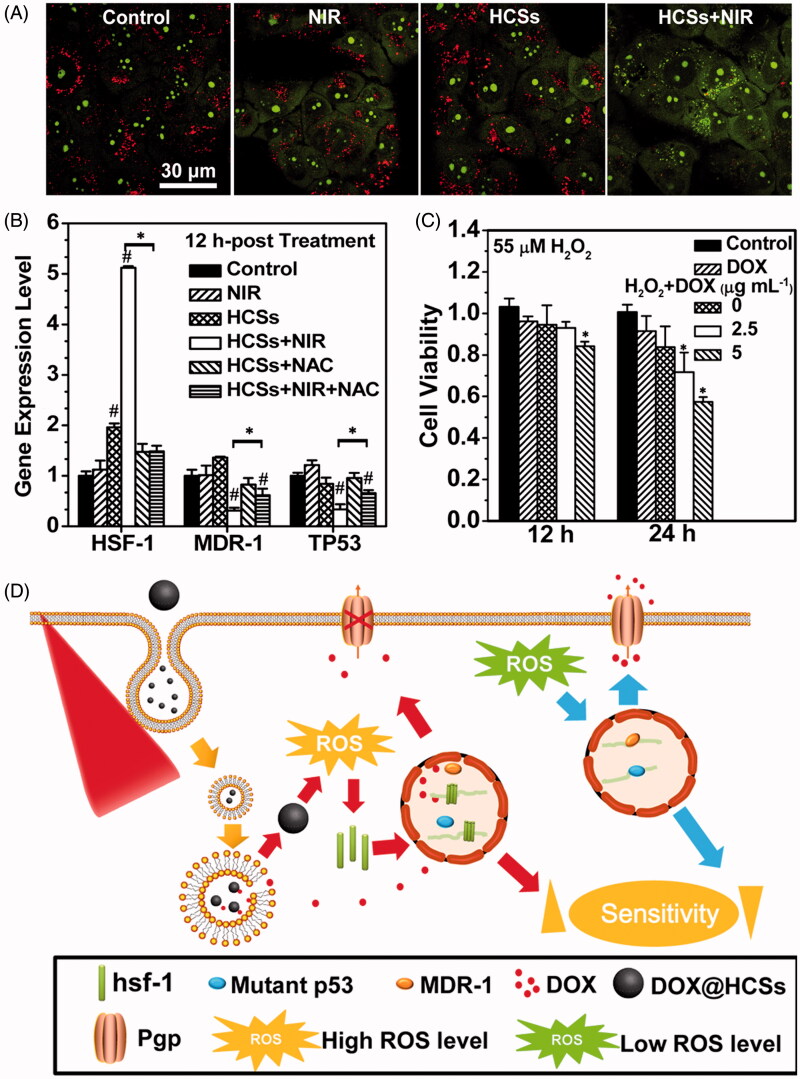
(A) Changes in the lysosomal membrane permeation induced by the photothermal effects determined by AO staining. (B) Impact of intracellular ROS levels on the expression of HSF-1, MDR-1 and TP53 genes. The asterisk (*) shows significant differences between test samples and the sample under laser irradiation (HCSs + NIR) (*p* < .05); the pound symbol (#) indicates significant differences between test samples and the control. (C) Change in the DOX sensitivity of MCF-7/ADR cells treated with 55 μM H_2_O_2_. Asterisk (*) indicates significant differences between control and test samples. (D) Combatting the chemotherapeutic resistance of cancer using HCSs under NIR laser irradiation. (Reprinted with permission from (Wang et al., 2015c), copyright 2015 American Chemical Society).

Wang et al. (2015c) have detailedly illustrated the mechanism of improved therapeutic efficacy by PTT using hollow carbon nanospheres (HCSs) as a carrier. When it comes to photothermal effects, (1) small blisters induced by the laser with high power-density could rupture the cell membrane and leads to cell death consequently; (2) lysosomal membrane permeation (LMP) could be increased which would allow for the escape of DOX@HCSs from lysosomes and facilitate the penetration of DOX into the nuclei. (3) The laser-converted heat could render DOX easily dissociate from the carbon matrix, thus prevent incomplete drug release at the diseased site (Figure 3(A)). While as for combatting MDR under laser irradiation, (1) higher DOX accumulation inside the nucleus was achieved under NIR irradiation due to the elevated expression of an HSF-1 gene which could encode a large amount of HSF-1 protein homotrimers to suppress the resistance-related pathways (Figure 3(B,C)); (2) high level of ROS could be induced by MCNs under NIR irradiation to sensitize the MCF-7/ADR cells to DOX. As a result, the HCSs were used to combat chemoresistance using synergistic chemotherapy and laser irradiation stimuli to produce heat and free radicals (Figure 3(D)). These HCSs had a high load efficiency and can deliver a large amount of drugs into cells. In addition, laser irradiation of HCSs can not only induce photothermal effects, but also disrupt intracellular redox state, which released persistent free radicals. The free radicals will improve HSF-1 gene expression and promote the production of hsf-1 protein to suppress resistance-related pathways. The viability of resistant cells was reduced since laser excitation of DOX@HCSs could not only release DOX but also serve as a ROS generator by triggering the generation of lots of free radicals.

Carbon materials alone could serve as carriers for drug delivery and combine with other materials to form hybrid composites. For example, mesoporous silica coated carbon (carbon-silica nanocomposite) with improved the biocompatibility and functionalizable surface were synthesized and used for drug delivery. Wang et al. (2014c) had designed a core-shell graphitic carbon@silica nanospheres system, which possessed both high drug-loading capacity and controllable drug release pattern. The semigraphitized carbon exhibited many favorable properties: (1) high drug-loading capacity due to the sp2-hybridized framework, (2) better photothermal conversion capacity due to the hotspots of the graphitic pore walls. Moreover, the mesoporous silica shell could be modified to ensure hydrophilicity and targeted drug delivery. In addition, Wang et al. (2015b) conjugated a new HB5 aptamer on the DOX-loaded mesoporous carbon–silica composite (MSCN-PEG/DOX), which could target the HER2-overexpressed breast cancer cells (SK-BR-3). The combination index (CI) was 0.253, indicating the synergistic effect of chemotherapy and photo-thermal-therapy of MSCN-PEG-HB5/DOX. Another study reported by Zhang et al. (2015a), in which MCNs with a diameter of 150–200 nm were capped with the copper sulfide (CuS) nanoparticles (NPs). CuS was a well-known p-type semiconductor material and can be explored to promote the NIR absorption ability and photothermal conversion ability.

### 3.2. Therapeutic biomolecule delivery

**Figure 4.**
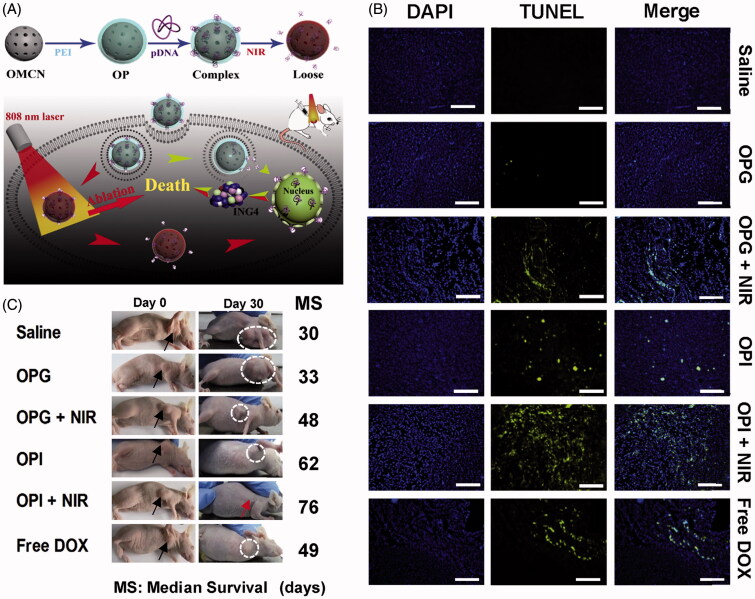
(A) Illustration of the synthesis and combined photothermal combined gene therapy achieved by PEI-grafted OMCN. (B) Apoptosis results on 14th day postinjection with different treatments based on TUNEL assay. Blue: DAPI-stained nucleus; green: FITC-labeled apoptosis cells. (C) Images of mice on 30th day postinjection and median survival data with different treatments. Black arrows and white dotted circles indicated the tumor sites before and after treatments, respectively. (Reproduced with permission from (Meng et al., 2016), copyright by Elsevier).

Apart from delivering traditional chemical drugs, MCNs could also be explored to transport macromolecules, such as genes or proteins. Gene therapy is thought to be an effective and safe way to suppress oncogenes and restrain the proliferation of intractable tumors through developing exogenous nucleic acids as therapeutic agents. In order to address the challenges including easy degradability and poor cellular uptake efficiency of nucleic acids, Meng et al. (2016) designed a PEI-modified oxidized mesoporous carbon nanosphere (OP) for combined photothermal and gene therapy (Figure 4(A)). The synthesized OP had three-dimensional spherical structure with a uniform diameter, ordered mesopores with graphitic domains, excellent water dispersibility and good biocompatibility. Plasmid DNA that could encode ING4 and pING4 was chosen to be loaded into OP to suppress the cancer cell proliferation and tumor angiogenesis. The prepared OP was exploited as both the photothermal carrier with strong NIR light absorption ability and the gene vector via electrostatic attraction. This system could not only deliver the therapeutic gene to tumor tissue for gene therapy, but also eliminate the tumor by photothermal ablation. As shown in TUNEL assay (Figure 4(B)), compared with other groups, PTT combined with gene therapy induced most obvious and extensive apoptosis of tumor tissues. The result of the median survival data (Figure 4(C)) indicated that the mice received combinational photothermal and gene therapy had the median survival time of 76 days, which was much longer than those treated by PTT (48 days) or gene therapy alone (62 days). In addition, the tumor of some mice completely disappeared after the combinational therapy and stayed alive till the end of the experiment. Therefore, combining PTT with gene therapy could induce severe and extensive apoptosis of tumor cells, significantly inhibit the tumor growth, and extend the life span of the tumor-bearing mice.

The MCNs with the relatively large mesopores could not only deliver the nucleic acid for cancer treatment, but also load proteins and act as a novel kind of adjuvant for oral vaccine delivery. Wang et al. (2011) synthesized a kind of mesoporous carbon (C1) with a particle size of 470 nm by employing mesoporous silica as a template and sucrose as a carbon source. CI was expected to effectively prevent the degradation of vaccine in the gastrointestinal tract and be preferentially uptake by M cells. Herein, BSA was used as the model antigen to be loaded into the pores of C1. After oral immunization the Balb/c mice with BSA-loaded C1, the IgG titer reached to a level almost equal to that induced by the parenteral administrated antigen emulsified in Freund’s complete adjuvant (FCA). And the mucosal IgA was detected in intestinal, vaginal and salivary secretions, which suggested that the BSA-loaded C1 could effectively stimulate mucosal immune response. In addition, both T-helper 1 and T-helper 2 mediated responses were induced. These results demonstrated that the MCNs could be used as a novel vaccine adjuvant and exhibited great potential on the modulation of immune response.

### 3.3. Bioimaging

The increasing number of cancer-related cases and deaths around the world has spurred wide-spread attention to (1) targeted delivery of therapeutic agents, (2) early detection and diagnosis and (3) *in vivo* real-time monitoring of the therapeutic responses. The term ‘theranostic’ was introduced to combine these therapeutic and diagnostic agents into one system.

#### 3.3.1. MCN-assisted fluorescent imaging

**Figure 5.**
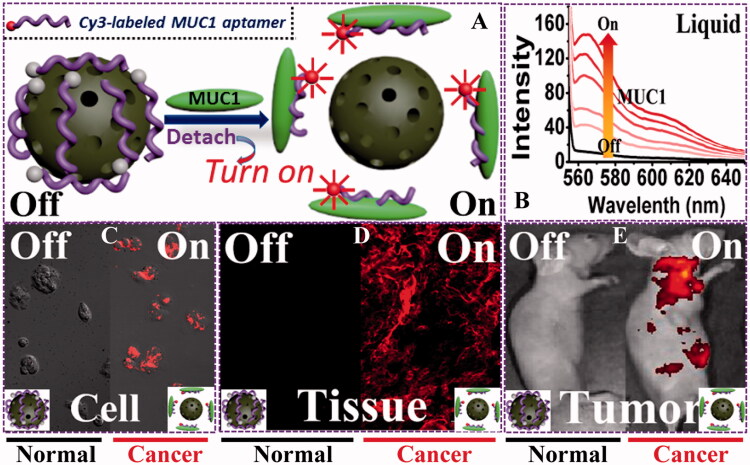
(A) Schematic illustration of the sensing principle based on the OMCN/P0-Cy3 aptasensor. (B) MUC1 responsive fluorescent recovery in the buffer solution recorded by fluorescence spectrophotometer. (C–E) Sensing performances of the OMCN/P0-Cy3 aptasensor in cell level (confocal fluorescence microscopy image), tissue level (inverted fluorescence microscopy image), and whole animal level (*in vivo* fluorescent image), respectively. (Reprinted with permission from (Li et al., 2015a), copyright 2015 American Chemical Society).

Mesoporous carbon nanomaterials have become a novel theranostic nano-platform due to their facile functionalization, good biocompatibility, and especially the supramolecular π–π stacking. Supramolecular π–π stacking can tentatively quench the fluorescence of the grafted or loaded fluorescent molecules attached to MCNs, and the fluorescence will recover once the molecules detached from the MCNs. Li et al. (2015a) modified a Cy3-labeled ssDNA probe (P0-Cy3) on the surface of oxidized mesoporous carbon nanospheres (OMCN) to create a fluorescent aptasensor. The aptasensor could detect the mucin1 protein in liquid with excellent selectivity, and quantify the cancer cells in solution. The spherical OMCN with well-defined mesoporous structure, hydrophilic carboxyl surfaces, and obvious graphitization domains could quench the fluorescence of P0-Cy3 by noncovalent π–π stacking, which was then quantificationally recovered once exposed to the targeted site (Figure 5(A)). It was found that the OMCN-based aptasensor could image cancer cells and solid tumors with high specificity (Figure 5(C–E)). The quenched fluorescence was clearly turned on in solid tumors, while the normal tissue showed no fluorescence. Therefore, it was easy to distinguish tumor tissue from normal tissue. Confocal laser scanning microscopy (CLSM) results confirmed the fluorescence ‘turn-on’ phenomenon. In MCF-7 cancer cells incubated with OMCN/P0-Cy3, enhanced fluorescence could be seen with the extension of incubation time while no fluorescence could be detected for the noncancerous cells.

Combining MCNs and carbon dots (CDs) has become a new *in vivo* real-time imaging technique. CDs exhibit multicolor and wavelength-dependent fluorescence upon laser excitation. The application of CDs as a drug carrier is still limited because of their nonporous structure and low drug loading capacity. Therefore, the combination of MCNs and CDs is necessary for biomedical application. Kong et al. (2014) prepared fluorescent MCNs through a simple precursor carbonization-in-hot solvent route. The BET surface area and pore volume of the MCNs were 864 m^2^g^−1^ and 0.91 cm^3^g^−1^, respectively. The particle size and pore size distribution of the MCNs were approximately 100 nm and 2.7 nm, respectively. The quantum yield of fluorescent MCNs was calculated to be ca. 37% under 360 nm excitation which is higher than that of most reported carbon dots (Bourlinos et al., 2008; Tian et al., 2009). In addition, MCNs could be endowed with multicolor and upconversion photoluminescence, which were similar to the properties of reported CDs (Liu et al., 2009). CLSM images indicated that cancer cells were illuminated after the endocytosis of fluorescent MCNs. Moreover, the cell-labelling fluorescence was photo-stable and exhibited better performance than organic fluorescent dyes. Similar results were reported by Wang et al. (2014b, 2015a). They fabricated fluorescent porous carbon nanocapsules (FPC-NCs) with fluorescent CDs embedded in the porous carbon shell. The incorporated CDs could be explored as an optical imaging contrast candidate for CLSM and two-photon fluorescence cell imaging.

#### 3.3.2. MCN-assisted MR imaging

Magnetic resonance (MR) imaging is a widely used biomedical tool that is capable to noninvasively obtain anatomic information with high spatial and temporal resolution (Hricak, 2005). The MRI imaging of MCNs was mostly realized by embedding certain inorganic NPs into the carbonaceous structure, such as adolinium (Gd) chelates (Zhang et al., 2017b), Fe_3_O_4_ and manganese oxide (Zhang et al., 2015b). Zhang et al. (2015b) developed an intelligent stimuli-responsive nano-system based on MCNs to improve the resolution and specificity of diagnostic imaging and enhance the therapeutic efficiency for cancer treatment. By taking advantage of the “breaking up” nature of manganese oxides (MnOx) and the specific interaction between the carbonaceous framework and aromatic drug molecules, MnOx NPs were integrated into HMCNs by a simple in situ redox strategy. The nanoparticles could then be used in pH-sensitive T1-weighted MR imaging and pH/HIFU dual-responsive on-demand drug release. The ultrasensitive disease-triggered MR imaging performance demonstrated in 4T1 xenograft nude mice and by a 52-fold increase in longitudinal relaxivity. In addition, the inorganic NPs helped minimize metastasis while maintaining low hemolysis percent and high histocompatibility.

#### 3.3.3. MCN-assisted photoacoustic imaging

Photoacoustic (PA) imaging is a powerful diagnostic imaging tool containing a pulsed laser as an energy source and ultrasonic waves as the signal (Pu et al., 2014; Zhang et al., 2014c). When a pulsed laser encounters tissues and other bodily components, the light energy is changed to heat, and the temperature of the targeted region increases. The increase in temperature results in thermo-elastic expansion that generates ultrasonic waves. Compared with the traditional optical imaging methodologies, PA imaging has higher spatial resolution and sensitivity, and deeper tissue penetration. These improvements are in part caused by detection of phonons, a collection of vibrational motions, instead of photons, packets of energy. Phonons cannot be scattered in tissues like photons (Pu et al., 2014). Lee’s group (Zhang et al., 2017a) designed a degradable hollow mesoporous PEG-Si/C NP for pH-responsive, photoacoustic imaging-guided chemo-thermal combination therapy. Porous silicon nanostructures show potential for multiple bioapplications due to their intrinsic large cavity, biodegradability and biocompatibility (Alhmoud et al., 2015; Shrestha et al., 2016). Carbon-based nanomaterials could also have potential since they have excellent NIR absorption, biocompatibility and high photostability (Wang et al., 2014c, 2015a). By combining the two materials, the Si/C NPs were used as an efficient and biodegradable PTT carrier showing good PA signal. Selective accumulation of PEG-Si/C-DOX around tumor tissue was observed with photoacoustic images. PEG-Si/C-DOX NPs achieved complete in vivo tumor elimination via combinational chemo-thermal therapy.

## 4. Conclusions and outlook

In this review, we summarized the major advances of MCNs involving drug delivery and biomedical applications. MCNs hold great potential as a drug carrier to modulate drug release and realize spatio-temporal drug delivery due to a large surface area and pore volume, adjustable pore structure and easily-modified surface. Moreover, due to the capacity of MCNs to convert NIR light to heat, the combinational PTT and chemotherapy could be achieved to improve the clinical therapeutic effect. After incorporating with fluorescent dyes or carbon dots, MCNs can be used for bio-detection and real-time imaging. Therefore, MCNs are considered as the next generation of platforms for drug delivery and biomedical applications.

However, the biomedical applications of MCNs are still at the infancy stage. There still remain several significant challenges and concerns regarding the diseases treatment based on MCNs, which need to be addressed in the practical applications. For example, the *in vivo* real-time monitoring of MCNs vehicles *via* bioimaging method needs to be developed. More detailed knowledge about drug delivery systems and biomedicine based on MCNs is needed to develop more comprehensive therapeutics and diagnostics in the field of nanomedicine. More versatile but straightforward nanomaterials with efficient, controllable drug release pattern or combining with other treatments need to be designed for practical applications. In addition, most of the reports about the toxicological data are based on the *in vitro* tests on the cell level, our knowledge about the in vivo fates and potential long-term toxicity is still limited due to the insufficient data and discrepancies of between the *in vitro* and *in vivo* results. Whether and how those nanomaterials will affect the immune systems, nerve systems and reproductive systems, have not yet been investigated systematically, and therefore, intensive study about the *in vitro* or *in vivo* toxicity and distribution of carbon based material is necessary (Cheung et al., 2010; Zhang et al., 2010a; Liu et al., 2011b, 2013a; Yang et al., 2012b). Overall, to create mesoporous carbon vehicles that are suitable for clinical medical applications without safety concerns, more extensive and deeper *in vivo* test is necessary for this newly and rapidly developing field of nanomedicine.
